# Kidney lipid metabolism: impact on pediatric kidney diseases and modulation by early-life nutrition

**DOI:** 10.1007/s00467-024-06595-z

**Published:** 2024-11-27

**Authors:** Eva Nüsken, Jenny Voggel, Leon Saschin, Lutz T. Weber, Jörg Dötsch, Miguel A. Alejandre Alcazar, Kai-Dietrich Nüsken

**Affiliations:** 1 Clinic and Polyclinic for Pediatric and Adolescent Medicine, Faculty of Medicine and University Hospital Cologne, University of Cologne, Kerpener Str. 62, 50937 Cologne, Germany; 2 Center for Molecular Medicine Cologne (CMMC), University of Cologne, Cologne, Germany; 3 Cologne Excellence Cluster on Cellular Stress Responses in Aging-Associated Diseases (CECAD), University of Cologne, Cologne, Germany; 4 Institute for Lung Health, University of Giessen and Marburg Lung Center (UGMLC), Member of the German Center for Lung Research (DZL), Giessen, Germany

**Keywords:** Kidney lipid metabolism, Children, Early-life nutrition, Lipidomics, Oxidative stress, Antioxidants

## Abstract

**Abstract:**

Our review summarizes and evaluates the current state of knowledge on lipid metabolism in relation to the pathomechanisms of kidney disease with a focus on common pediatric kidney diseases. In addition, we discuss how nutrition in early childhood can alter kidney development and permanently shape kidney lipid and protein metabolism, which in turn affects kidney health and disease throughout life. Comprehensive integrated lipidomics and proteomics network analyses are becoming increasingly available and offer exciting new insights into metabolic signatures. Lipid accumulation, lipid peroxidation, oxidative stress, and dysregulated pro-inflammatory lipid mediator signaling have been identified as important mechanisms influencing the progression of minimal change disease, focal segmental glomerulosclerosis, membranous nephropathy, diabetic kidney disease, and acute kidney injury. We outline key features of metabolic homeostasis and lipid metabolic physiology in renal cells and discuss pathophysiological aspects in the pediatric context. On the one hand, special vulnerabilities such as reduced antioxidant capacity in neonates must be considered. On the other hand, there is a unique window of opportunity during kidney development, as nutrition in early life influences the composition of cellular phospholipid membranes in the growing kidney and thus affects local signaling pathways far beyond the growth phase.

**Graphical Abstract:**

**Figure:**
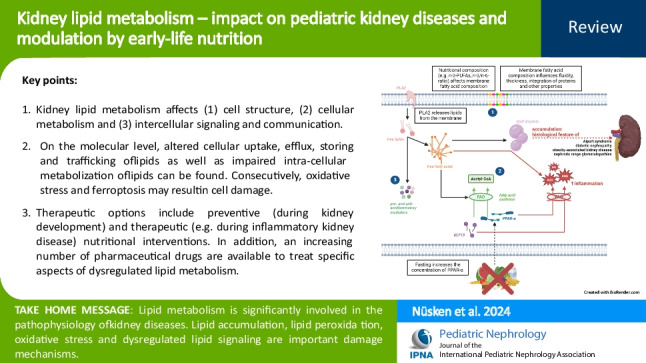
A higher resolution version of the Graphical abstract is available as Supplementary information

**Supplementary Information:**

The online version contains supplementary material available at 10.1007/s00467-024-06595-z.

## Introduction

In the early twentieth century, the term “lipoid nephrosis” was used for a clinical entity that we now refer to as minimal change disease (MCD) or, synonymously, as minimal change glomerulopathy with nephrotic syndrome. This historical context demonstrates that the role of dysregulated kidney lipid metabolism in pediatric kidney disease has been acknowledged for a long time [1]. Idiopathic nephrotic syndrome due to MCD is the most common glomerular disease in children [2]. The name “lipoid nephrosis” was derived from the observation that lipoid droplets were not only present in convoluted tubules on biopsy but also in urine, demonstrating that urine can also provide diagnostic access to kidney lipid metabolism [3]. Today our knowledge of MCD has significantly improved, with major advancements in the patients’ treatment [2]. However, renal lipid metabolism has only recently gained attention both scientifically and clinically. It is now well established that impaired renal lipid metabolism plays a central role in the progression of MCD, focal segmental glomerulosclerosis (FSGS), acute kidney injury (AKI), membranous nephropathy, and diabetic kidney disease.

**Fig. 1:**
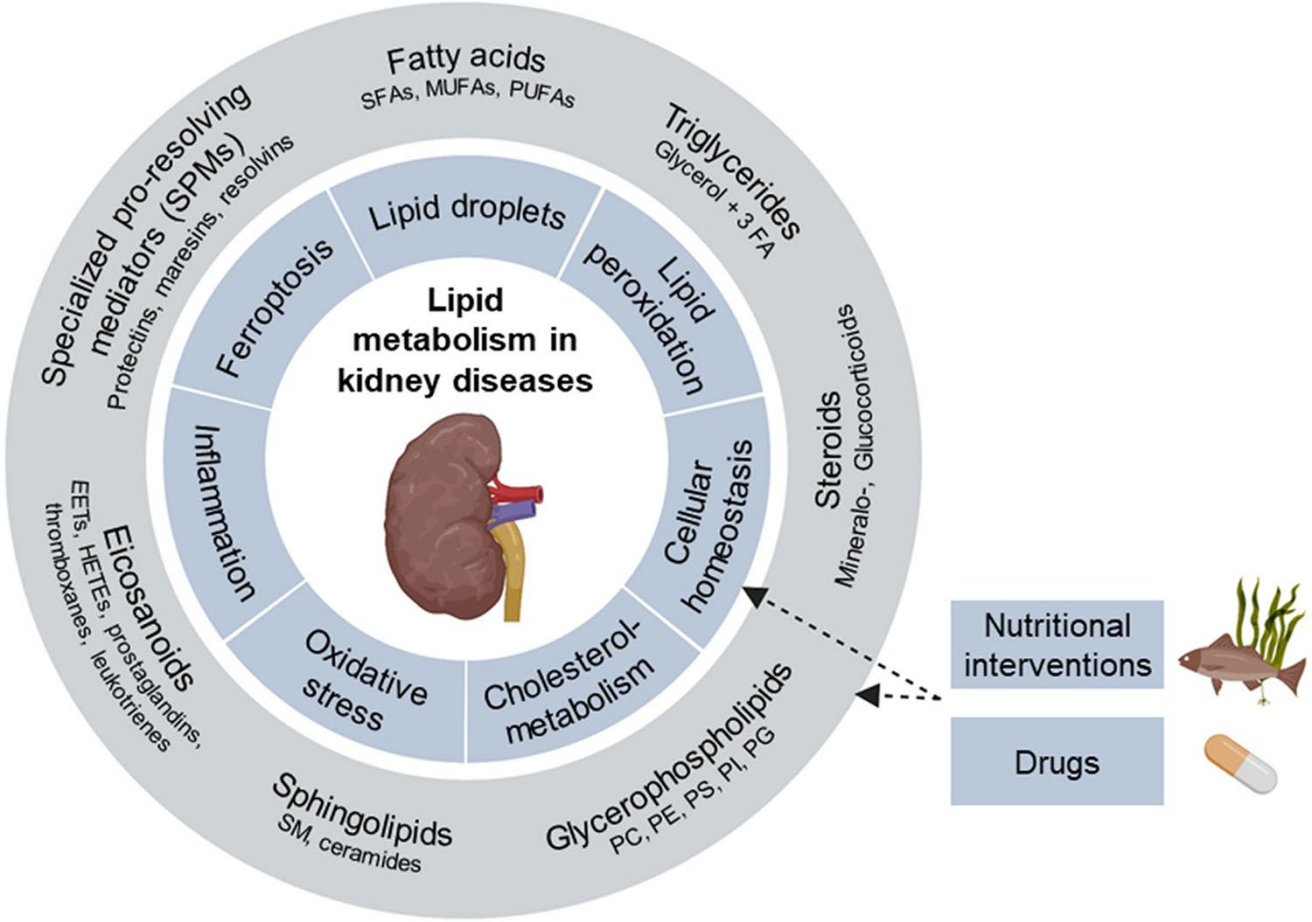
An overview of the lipid classes and associated pathways and categories in kidney diseases. SFAs, saturated fatty acids; MUFAs, monounsaturated fatty acids; PUFAs, polyunsaturated fatty acids; FA, fatty acids; PC, phosphatidylcholine; PE, phosphatidylethanolamine; PS, phosphatidylserine; PI, phosphatidylinositol; PG, phosphatidylglycerol, SM, sphingomyelin, EET, epoxyeicosatetraenoic acid; HETE, hydroxyeicosatetraenoic acid. Created with BioRender.com

Our review aims to summarize and evaluate current knowledge on lipid metabolism in relation to the pathomechanisms of kidney disease (Fig. 1) with a focus on common pediatric kidney diseases. In addition, we highlight the role of nutrition throughout kidney development and growth, i.e., from intrauterine life to adolescence, in the modulation of kidney lipid metabolism, in turn affecting kidney health and disease throughout life.

### Kidney lipid metabolism: diagnostic tools and clinical relevance

While assessment of steatosis in liver tissue is a routine analysis, it is not routinely performed in kidney biopsies, which may contribute to underestimation of impaired renal lipid metabolism. In the kidney, lipid droplet accumulation can be visualized and quantified, e.g., by electron microscopy or Oil Red O and BODIPY staining [4]. Using these techniques, it has become evident that intracellular lipid droplet accumulation is a histologic feature of many different kidney diseases like diabetic nephropathy [4], obesity-associated kidney disease, nephrotic-range glomerulopathies [5], or Alport syndrome [6]. The localization of steatosis is related to the underlying pathophysiological processes. Thus, membranous nephropathy typically leads to glomerular lipid deposition [7, 8] whereas ischemic kidney injury is more likely to induce tubular lipid accumulation [9]. Diabetic kidney disease can be associated with both glomerular and tubular lipid deposition [4].

Since multi-omics analyses of urine, urinary exosomes, and urinary kidney cells (“liquid biopsy”) are currently being established as diagnostic tools [10], integrated network analyses will provide deeper insights into the molecular basis of impaired kidney lipid metabolism [11]. With growing numbers of clinical studies using lipidomics, specific lipid mediators have already been recognized as possible predictors of disease progression or new therapeutic targets. In diabetic kidney disease for example, levels of urinary lysophosphatidylcholines containing either saturated palmitic (C16:0) or stearic (C18:0) acid correlated with glomerular filtration rate (GFR) decline [12].

## Lipid components and their physiological homeostasis

### Membrane lipids impact upon the properties of membranes

Glycerophospholipids are essential components of cell membranes and their composition can be influenced by dietary intake of lipids [11, 13]. Phospholipase A2 (PLA2) enzymes are responsible for releasing fatty acids from glycerophospholipids, which can then be used as substrates for the production of pro- and anti-inflammatory lipid mediators [14]. In humans, there are different PLA2 subtypes which differ in cellular localization and substrate specificity and activity [14]. The composition of membrane fatty acids affects the properties of membranes, such as fluidity and integration of proteins, which in turn affect cell function. Thus, supplementation with eicosapentaenoic acid (EPA) can impact B cell differentiation and alleviate autoimmune diseases by modulating membrane fluidity [15].

Sphingolipids are another important component of cell membranes and serve as both structural components and sources of signaling molecules [16]. It has been suggested that the sphingolipid pathway may protect cells from excessive fatty acids, as ceramides promote the utilization of fatty acids for energy and decrease mitochondrial efficiency [17]. Activation of the S1P receptor 1 is important for recovery from AKI. However, high sphingosine levels may also contribute to glomerular diseases including diabetic kidney disease and FSGS [18, 19].

### Receptors and transport proteins regulate cellular uptake of lipids

**Fig. 2:**
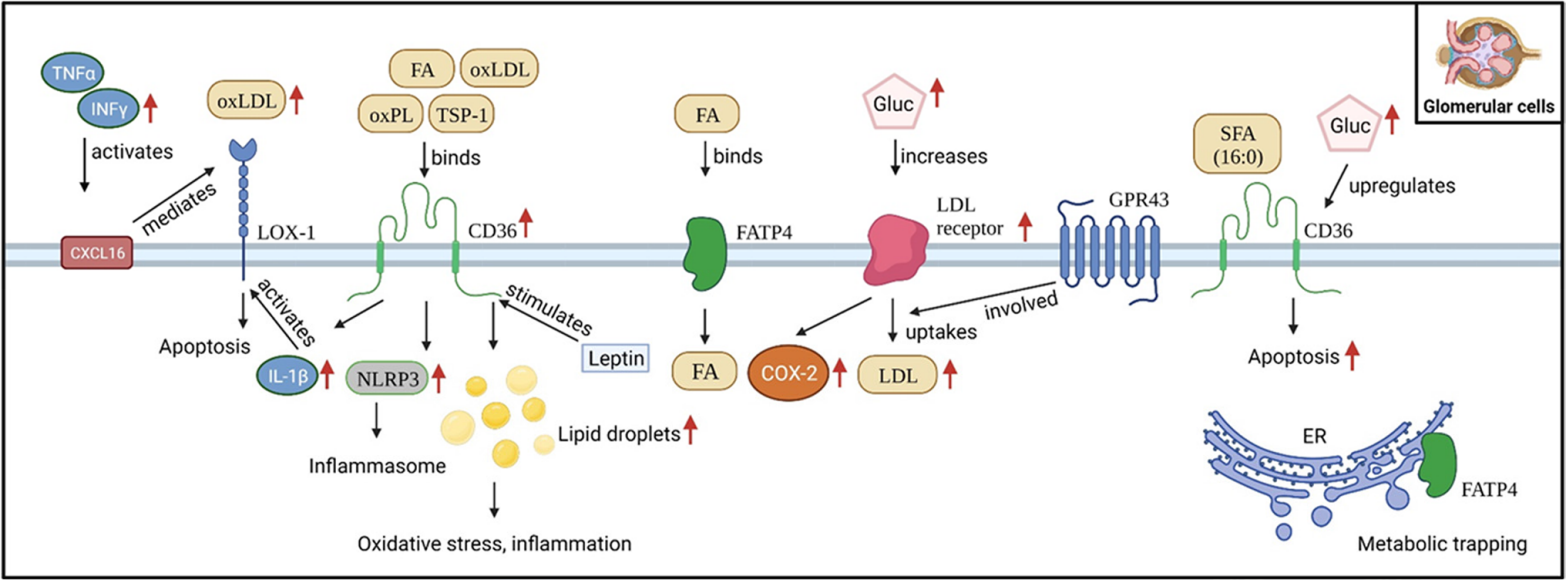
Receptors and transport proteins involved in lipid uptake of glomerular and tubular cells. TSP-1, thrombospondin 1; GPR43, G protein-coupled receptor 43; FA, fatty acids; NLRP3, NLR family pyrin domain containing 3; FATP4, fatty acid transport protein 4; CXCL16, scavenger receptor for phosphatidylserine, and oxidized low-density lipoprotein. Created with BioRender.com

Cell type-specific sets of receptors and transport proteins (Fig. 2) regulate the uptake of lipids from both the blood and—in tubular cells—from the urine. Recently, uptake of oxidized lipids has been attributed a particularly important pathophysiological role. On the one hand, oxidized low-density lipoprotein (oxLDL) can contribute to cell death of, e.g., podocytes via the activation of the NLRP3/inflammasome cascade [20]. On the other hand, it can promote glomerulosclerosis by stimulating fibronectin expression [21]. In the following, we present disease-relevant receptors and transport proteins for the most important kidney cell types.

#### Podocytes

CXCL16 acts as a scavenger receptor that is responsible for the cellular uptake of oxLDL. Increased expression of CXCL16 has been observed in diseases such as primary nephrotic syndrome and membranous nephropathy [7, 22]. Blocking CXCL16 reduces the uptake of oxLDL by podocytes, while stimulation with cytokines like TNF-α or IFNγ increases CXCL16 expression [22]. CD36, on the other hand, binds not only oxidized phospholipids but also fatty acids and other molecules like thrombospondin 1 [23]. Leptin, an adipose tissue-derived hormone involved in appetite regulation, has been identified as a key stimulator for CD36 expression in podocytes [24]. Another important regulator of lipid uptake in podocytes is fatty acid transport protein 4 (FATP4). Its upregulation in diabetic kidney disease leads to lipid accumulation and disease progression [25]. Lately, other receptors, such as GPR43, have been identified to be involved in lipid accumulation in diabetic kidney disease [26].

#### Mesangial cells

Stimulation with saturated fatty acids upregulates CD36. Blocking of CD36 reduces cellular lipid accumulation and decreases markers of oxidative stress and inflammation [27]. Stimulation with IL-1β upregulates lectin-like oxLDL receptor 1 (LOX-1) expression and concomitantly increases cellular oxLDL uptake [28].

#### Proximal tubular cells (PTCs)

PTCs use fatty acids as primary source of energy to cover their high baseline level of ATP consumption [29]. Stimulation of HK-2 cells with high glucose conditions increases CD36 expression and induces epithelial-to-mesenchymal transition which can be prevented by blocking CD36 [30]. Albumin-bound non-esterified fatty acids can be taken up from the luminal side using fatty acid transport protein 2 (FATP2) [31]. Uptake of fatty acids from the urine is especially relevant in proteinuric kidney disease when high amounts of albumin-bound fatty acids are excreted. In prior mouse experiments, urinary excretion of albumin loaded with free fatty acids resulted in aggravated tubulointerstitial damage compared to urinary excretion of albumin alone [32].

### Free fatty acids are an important energy source but can also promote oxidative stress

Fatty acid oxidation (FAO) is the most efficient method for providing energy in cells with high metabolic activity such as proximal tubule cells [29]. Rate-limiting enzymes involved in FAO [e.g., carnitine palmitoyltransferase I (CPT-1) and acetyl-CoA acyltransferase 2 (ACAA2)] are regulated by transcription factors like peroxisome proliferator-activated receptor alpha (PPAR-α) and Krüppel-like factor 15 (KLF15) [33–36]. Fasting-induced upregulation of PPARs is associated with increased expression of genes involved in FAO [37] whereas loss of PPAR-α decreases FAO [34, 35]. Loss of KLF15 in proximal tubular cells impairs FAO and exacerbates tubular damage and fibrosis [36]. In addition, activation of AMP-activated protein kinase (AMPK) during reduced energy availability also promotes FAO [38] and suppresses lipid biogenesis [39]. Under physiological conditions, free fatty acids (FFA) that are not required are stored in lipid droplets [33] or further metabolized. Excess FFAs due to defective FAO, defective lipid droplet formation or increased availability may lead to lipotoxicity by promoting reactive oxygen species (ROS) production, endoplasmic reticulum (ER) stress, and inflammation [33]. Kidney cells are particularly susceptible to FFA-induced lipotoxicity [40]. Omega-3 polyunsaturated fatty acids (n-3 PUFAs) like docosahexaenoic acid (DHA) seem to inhibit proliferation of mesangial cells [41]. However, recent studies suggest that the proliferation-inhibiting effect of DHA may not be due to the native form of DHA, but to the oxidized form (oxDHA), which leads to increased intracellular ROS levels and subsequent activation of apoptosis [42].

### Signaling molecules derived from arachidonic acid are important for normal kidney development but are also involved in inflammation and arterial hypertension

**Fig. 3:**
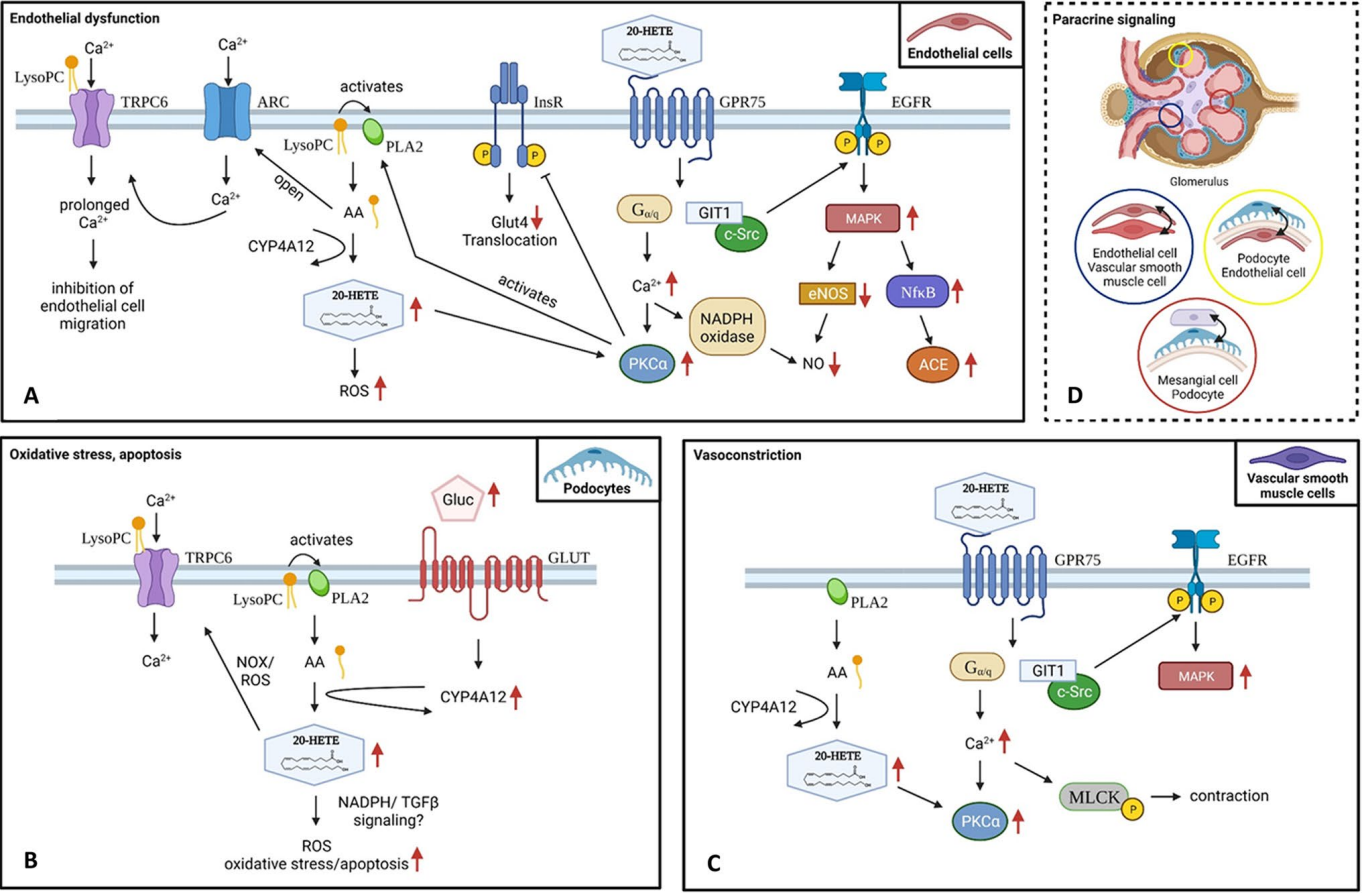
Roles of 20-HETE in different glomerular cell types. Activation of 20-HETE due to increased CYP-mediated AA metabolism can lead to **A** endothelial dysfunction, **B** oxidative stress and apoptosis, as well as **C** vasoconstriction; **D** paracrine signaling could be of major importance. AA, arachidonic acid; ACE, angiotensin converting enzyme; ARC, arachidonate-regulated calcium channels; CYP, cytochrome P450; CYP4A12, cytochrome P450 A12; c-Src, tyrosinkinase Src; EGFR, epidermal growth factor receptor; GIT1, ARF GTPase-activating protein; Gluc, glucose; GPR75, G-protein coupled receptor 75; 20-HETE, 20-hydroxyeicosatetraenoic acid; InsR, insulin receptor; MLCK, myosin light-chain kinase, PKCα, protein kinase alpha; PLA2, phospholipase A2; ROS, reactive oxygen species; TRPC6, transient receptor potential 6. Created with BioRender.com

Arachidonic acid (AA) is the most prevalent omega-6 polyunsaturated fatty acid (n-6 PUFA) in mammals and is metabolized by different cytochrome P450 (CYP) enzymes into epoxyeicosatrienoic acids (EETs) and hydroxyeicosatetraenoic acids (HETEs). Importantly, AA-metabolizing CYP enzymes differ between species, which is relevant for translational research [43]. EETs mediate vasodilatation, inhibition of inflammation, and excretion of sodium. Therefore, soluble epoxide hydrolase (sEH) inhibitors and EET analogs have been studied as anti-hypertensive drugs [44]. The CYP4A-derived metabolite 20-HETE contributes to vascular dysfunction and oxidative stress in pre-glomerular arteries and promotes vascular remodeling [45]. Lately, evidence is accumulating that 20-HETE also has a role in podocyte pathology [46]. Most interestingly, it has been shown that 20-HETE can activate transient receptor potential-6 channels (TRPC6) in podocytes [46] which are required for maintenance of podocyte structure and function [46]. Our current understanding of 20-HETE signaling in glomerular cells is presented in Fig. 3.

Via cyclooxygenases 1 and 2 (COX-1 and COX-2), AA is metabolized into prostaglandin H2 (PGH2), a precursor of other prostaglandins and thromboxane. Prostaglandin E2 (PGE2) is a well-known lipid mediator derived from PGH2 with relevance in tubular and podocyte disease [47]. In experimental nephrotoxic serum nephritis, blockade of PGE2 mitigated the inflammatory response [48]. In neonatal nephrology, tubular dysfunction in antenatal Bartter syndrome (i.e., “hyperprostaglandin E syndrome”) can be significantly ameliorated by nonsteroidal anti-inflammatory drugs (NSAIDs), which emphasizes the pivotal role of PGE2 in tubular function [49]. However, during embryonic development, PGE2 is needed for ciliogenesis and impaired availability of PGE2 may compromise kidney development [50]. Prostacyclin (PGI2) is a PGH2-derived metabolite mainly produced in endothelial cells which mediates vasodilation. There is evidence that PGI2 may decrease the susceptibility to AKI by hypotensive events and protect the kidney from ischemia reperfusion injury [51]. On the other hand, PGI2 may exert adverse effects when inadequate dilation of preglomerular arterioles occurs in hypertensive individuals [52].

Finally, AA can be metabolized by lipoxygenases (LOX) into leukotrienes (LTs) and lipoxins (LXs), which are important pro-inflammatory chemokines. Further metabolism results in production of HETEs, like in the CYP pathway [53].

### Signaling molecules derived from omega-3 PUFAs have mainly anti-inflammatory effects

Docosahexaenoic acid (DHA; C22:6), eicosapentaenoic acid (EPA; C20:5), and omega-3 docosapentaenoic acid (n-3 DPA; n-3 C22:5) are the most important n-3 PUFAs in human physiology. Circulating n-3 PUFAs originating from seafood are associated with a lower incidence of chronic kidney disease (CKD) [54]. N-3 PUFAs can be metabolized into bioactive lipid mediators via COX, LOX, and CYP enzymes [55]. Eicosanoids derived from n-3 PUFAs act antagonistically to AA-derived eicosanoids on various levels. Thus, when being metabolized by COX, EPA is converted into prostaglandin E3 (PGE3), which exerts anti-inflammatory effects by inhibiting M1-like polarization and promoting M2-like polarization of macrophages [56]. Via the LOX pathway, EPA and DHA can be metabolized into “specialized pro-resolving mediators.” This class of lipid mediators has an important role in actively limiting inflammatory processes while enhancing microbial clearance and promoting organ recovery at the same time [57]. According to what is known so far, they bind to G protein-coupled receptors (GPCRs) like ALX and GPRs 18, 32, and 120 [55]. Additionally, EPA is converted via LOX into hydroxyeicosapentaenoic acids (HEPEs), a class of lipid mediators which is known to activate PPARs and thereby decrease lipotoxicity [55]. 12-HEPE can prevent foamy transformation of macrophages and ameliorate atherosclerosis [58]. Metabolism of DHA via CYP pathways promotes the formation of epoxydocosapentaenoic acids (EDPs) and dihydroxydocosapentaenoic acids (DiHDPAs). Despite our knowledge on biochemical pathways, the complex pathophysiological roles of n-3 PUFAs and their metabolites are still insufficiently understood. For example, and contrary to expectations, the DHA-metabolite 19,20-EDP significantly reduced survival rates in murine ischemia–reperfusion injury, while 14(15)-EET, the major epoxide metabolite of n-6 PUFA AA, had beneficial effects [59].

### Lipid peroxidation, oxidative stress, and ferroptosis are important pathomechanisms involved in kidney damage

Lipids and especially PUFAs are very susceptible to peroxidation [60]. Oxidative stress by accumulation of lipid peroxides can induce ferroptosis [61], which has been identified as an important iron-dependent mechanism of cell death contributing to AKI [62, 63] and diabetic nephropathy [64]. Recent studies confirm that peroxisomes are critically involved in this process. On the one hand, they contribute to removal of ROS [65]. On the other hand, they synthesize polyunsaturated ether-linked phospholipids which are even more sensitive to peroxidation and thus promote ferroptosis [66]. Dysfunction of glutathione peroxidase 4 (GPX4), which protects cells from oxidative stress and is a key regulator of ferroptosis, leads to accumulation of lipid hydroperoxides and hypersensitizes mice to ferroptosis-associated ischemia–reperfusion injury [62]. In tubular cells, HIF-1 [9, 67, 68] and AMPK signaling [69] regulate lipid turnover and cellular concentrations of fatty acids, thereby also influencing oxidative stress levels. Thus, energy stress leading to AMPK-activation inactivates acetyl-CoA carboxylase (ACC) and decreases biosynthesis of PUFAs, going along with reduced peroxidation and ferroptosis [39]. In contrast, downregulation of PPAR-γ in tubular cells leads to downregulation of uncoupling protein 1 (UCP-1) which in turn increases the production of ROS [70]. Furthermore, elevated oxygen concentrations can cause IL-6-related inflammation and mitochondrial dysfunction not only in the lung but also in the kidney [71].

Finally, oxidative stress enhances the production of oxidized low-density lipoprotein (oxLDL). Increased uptake of oxLDL in kidney cells is mediated by pro-inflammatory cytokines like TNF-α [7] and IL1-β [28] which in turn may cause further inflammasome activation [20] and promote glomerulosclerosis [21].

### Lipid droplets not only store energy but may also protect against toxic free fatty acids

The accumulation of lipid droplets is a histopathological feature of various kidney diseases [1, 3, 4, 6, 9]. These droplets, which contain neutral lipids such as cholesterol esters and triacylglycerols, are formed by the endoplasmic reticulum and play a role in energy storage, membrane remodeling, and lipid transport [72, 73].

In times of hypoxia, the formation of lipid droplets is believed to have a protective effect. Inhibition of prolyl hydroxylase 2, an enzyme involved in the degradation of hypoxia-inducible factor, has been found to increase the expression of genes associated with lipid droplets (*plin2*, *plin4*, and *hilpda)* and enlarge the droplets in tubular cells. The authors suggested that this could be a mechanism to protect against toxic free fatty acids during hypoxic tissue damage [68]. Furthermore, in persons with genetic variants of apolipoprotein L1, it has been shown that decreased formation of lipid droplets might contribute to increased risk of FSGS [74]. Research on *Drosophila melanogaster* has shown that reducing the activity of the enzyme DGAT1, which plays a role in triglyceride synthesis, leads to a decrease in lipid droplet formation and an increase in lipid peroxidation [75]. In contrast, nephrocyte overexpression of adipose triglyceride lipase (ATGL), an enzyme mediating fatty acid release from lipid droplets, rescued high fat diet-induced ER and mitochondrial dysfunction [75]. The authors suggest that the upregulation of ATGL may protect nephrocytes from high-fat diet-induced injury by directing fatty acids toward the mitochondria for beta-oxidation. Interestingly, fasting was found to upregulate ATGL expression in mouse kidney lysates, implying that the release of fatty acids from lipid droplets may be necessary to provide energy during this period [76]. Overall, maintaining lipid droplet homeostasis is crucial for kidney functioning.

### Impaired cellular lipid efflux may contribute to cell damage in kidney diseases

Cellular lipid accumulation arises not only from increased uptake or impaired cellular metabolism but also from decreased efflux. ATP-binding cassette transporter A1 (ABCA1) is an important transporter mediating cholesterol efflux from renal cells. Knockdown of ABCA1 in podocytes increased free cholesterol concentrations in the cytoplasm which was associated with cardiolipin accumulation and mitochondrial dysfunction [77]. Similarly, treatment of human podocytes with serum of diabetes mellitus patients or with TNF-α led induced cellular damage by reducing both the expression of the enzyme SOAT (Sterol-O-acyltransferase, mediating the formation of cholesterol esters) and ABCA1 [78]. In line with these in vitro data, ABCA1 was downregulated in kidney biopsies from patients with diabetic nephropathy [79].

## Kidney lipid metabolism: specific considerations in children

### Premature birth as a specific risk factor for renal oxidative stress

Clinical and experimental studies show that preterm infants are at higher risk for AKI [80] and CKD later in life. The risk to suffer from AKI is further increased by inborn errors of metabolism [81]. From a mechanistic point of view, oxidative stress during kidney development is of special relevance and has been identified as a promising target for pharmaceutical and non-pharmaceutical approaches to improve long-term renal outcomes [82]. It is well-known that premature infants have reduced anti-oxidant capacities [83]. In addition, many preterm infants require treatments like parenteral nutrition [84] or oxygen supply [85] which further increase the oxidative stress load. After perinatal asphyxia, the activity of paraoxonase 1 (PON1), an enzyme protecting against lipid oxidation, as well as oxidative stress markers in the blood, correlate with the occurrence of neonatal AKI [86]. In a small clinical study, polymorphisms in hemeoxygenase-1, another enzyme involved in antioxidant and anti-inflammatory processes in the kidney, have been associated with lower rates of AKI in preterm neonates [87]. As described above, oxidative stress in the kidney is closely linked to ferroptosis. Although no data on neonatal kidney injury is available to date, ferroptosis has been identified as a key mechanism in ischemia–reperfusion injury [88].

### Developmental aspects of kidney lipid metabolism

The concept of “Developmental Origins of Health and Disease” (DOHaD) suggests that influences during pregnancy and early childhood can impact the development and function of organs throughout life. Animal studies have shown that alterations in lipid composition of early-life diet can lead to changes in renal lipid metabolism later on [11]. Maternal overweight/obesity is an independent risk factor for childhood CKD [89]. Experimental studies have found higher levels of oxidative stress, inflammation, and fibrosis in the kidneys of offspring from obese mothers. The risk of kidney damage is exacerbated when an additional insult, such as diabetic nephropathy, occurs [90]. In addition, factors such as obesity, dietary fat intake, and metabolic changes are often already present in early childhood and are associated with oxLDL concentrations, glomerular filtration rate (GFR), and arterial blood pressure in children [123]. Understanding the molecular pathogenesis of diseases is crucial for early prevention. In this context, it is important to consider that kidney lipid metabolism is influenced by a complex metabolic-inflammatory network that includes other organs like adipose tissue. Adipose tissue not only stores fat but also releases pro-inflammatory factors and hormones that can impact kidney health. The molecular mechanisms underlying the increased risk for kidney disease are related to key metabolic regulators like SIRT1, pAMPK, or KLF15. In mice, overexpressing SIRT1 or treating with a SIRT activator reduced lipid accumulation, oxidative stress, and inflammation in the kidneys but had little effect on fibrosis and albuminuria [91]. High-fat maternal diet caused downregulation of KLF15 and pAMPK in growing mouse offspring’s kidneys [92], alterations which shift lipid metabolism toward less FAO and predispose toward lipid accumulation [36], finally leading to oxidative stress. Beyond dietary influences, adverse genetic predisposition regarding key components of kidney lipid metabolism may contribute to premature onset of kidney diseases as well. For example, expression levels of CXCL16 were higher in children with FSGS on biopsy than in children with MCD [22], a finding which deserves further studies. For an overview of the proteins relevant for lipid metabolism and oxidative stress, including their main functions and the main study results, please see Table 1.

**Table 1:** Proteins relevant for lipid metabolism and oxidative stress in human kidney diseases, rodent kidney disease models, and cell culture experiments

Research organism	Protein	Main protein function (UniProt)	Known disease context	Source
*Human*	HMOX1	Catalyzes the oxidative cleavage of heme	HMOX1 polymorphism is associated with less AKI in preterm children	[87]
	PON1	Mediates protection of low-density lipoproteins against oxidative modification	PON1 activity correlates with the occurrence of AKI after perinatal asphyxia	[86]
	CXCL16	Acts as scavenger receptor which binds to oxLDL	Increased in children with primary nephrotic syndrome (FSGS > MCD)	[22] [7]
	CD36/FAT	Binds long-chain fatty acids and facilitates their transport into cells	Increased in diabetic kidney disease; blocking of CD36 in mesangial cells reduces lipid accumulation, markers of oxidative stress, and inflammation	[23] [27]
	ABCA1	Catalyzes the translocation of specific phospholipids	Reduced in diabetic kidney disease. Knockdown in podocytes increases free cholesterol in cytoplasm	[77, 79]
*Rodents*	GPX4	Reduces phospholipid hydroperoxides even if incorporated in membranes and lipoproteins	Dysfunction hypersensitizes to ferroptosis-associated ischemia–reperfusion injury	[62]
	SIRT1	Regulator of metabolic stress responses, e.g., activation of FAO	Overexpression reduces renal lipid accumulation, oxidative stress, and inflammation	[91]
	KLF15	Transcriptional regulator, involved in podocyte differentiation	Reduced by high fat diet; reduced expressions shift lipid metabolism toward less FAO, predisposing to lipid accumulation, relevant in AKI/acute tubular damage	[36, 92]
	pAMPK	Energy sensor protein kinase, phosphorylates transcription factor		
	FATP4	Mediates the import of long-chain fatty acids into the cell by facilitating their transport across cell membranes	Upregulation (e.g., by VEGF-B) is associated with lipid accumulation and disease progression, e.g., in diabetic nephropathy	[25]
	LDLR	Binds LDL and transports it into cells by endocytosis	High glucose increases expression in podocytes; relevant in diabetic nephropathy	[26]
	FATP2	Mediates the import of long-chain fatty acids into the cell by facilitating their transport across cell membranes	Increased expression contributes to increased absorption of FA by tubular cells and progression of diabetic nephropathy	[31]
*Cell culture*	PHD2	Cellular oxygen sensor that catalyzes HIF degradation	Inhibition of PDH2 increases the expression of genes associated with lipid droplets, may thereby protect against hypoxic kidney damage	[68]
	PLA2	Hydrolyzes the ester bond of phospholipids, thereby releasing fatty acids	OxLDL stimulates group IVA PLA2 which releases AA; metabolization of AA to 12-HETE may stimulate fibronectin transcription; relevant in glomerulosclerosis	[14] [21]

*HMOX1*, heme oxygenase 1; *PON1*, paraoxonase 1; *CXCL16*, C-X-C motif chemokine ligand 16 (oxLDL-receptor); *CD36*/*FAT*, cluster of differentiation 36/fatty acid translocase; *ABCA1*, ATP-binding cassette transporter A1; *GPX4*, glutathione peroxidase 4; *SIRT1*, sirtuin 1; *KLF15*, Krüppel-like factor 15; *pAMPK*, phosphorylated AMP-activated protein kinase; *FATP4*, long-chain fatty acid transport protein 4; *LDLR*, low-density lipoprotein receptor; *FATP2*, long-chain fatty acid transporter protein 2; *PHD2*, prolyl hydroxylase 2; *PLA2*, phospholipase 2; oxLDL, oxidized low-density lipoprotein; *HIF*, hypoxia inducible factor; *AKI*, acute kidney injury; *FSGS*, focal segmental glomerulosclerosis; *MCD*, minimal change disease; *FAO*, fatty acid oxidation; *COX*-*2*, cyclooxygenase-2

### Nutrition as a key regulator of kidney lipid metabolism

In human breast milk, lipids are the predominant macronutrients and lipid composition varies a lot depending on maternal nutrition and maternal body pools [93]. In experimental models, dietary behaviors and the composition of fatty acids in diets determine the composition of phospholipids in cell membranes [11, 13] and the synthesis of eicosanoids [11]. An association of individual concentrations of saturated fatty acids, monounsaturated fatty acids, and PUFAs in cell membranes and the risk and severity of chronic kidney diseases and cardiovascular disease has been shown [94]. However, nutrition in early childhood not only provides energy and “building blocks” for growth, but can also temporarily or permanently “imprint” metabolic regulation [92, 95]. Thus, it has been shown that even the biophysical structure of lipid droplets in early-life nutrition impacts upon serum triglyceride, glucose, and lysophosphatidylcholine levels as well as body composition of adult rats [96, 97]. Since serum and urine levels of certain lysophosphatidylcholines have been associated with the course of diabetic kidney disease [12] and altered body composition is a known risk factor for type 2 diabetes, this further underlines the diversity of possible interactions between early-life diet and later kidney health. Even after kidney development is complete, diet can influence renal lipid metabolism. For example, fasting mice show increased tubular uptake of free fatty acids from the blood, which is associated with an increase in genes involved in fatty acid metabolism and fat storage, such as the lipid droplet proteins *Plin2* and *Plin5* [37]. At the same time, C16 acyl-carnitine, long-chain ceramides, and glycosphingolipids were reduced in kidney tissue [37].

However, most studies of dietary interventions with PUFAs targeting lipid metabolism have taken a therapeutic rather than a preventive or “imprinting” approach. In animal models, diet can easily be standardized and proof-of-principle studies have highlighted the potential of dietary interventions [15, 41, 98]. In a rat model of hypertension, an n-3 PUFA-enriched diet attenuated arterial hypertension induced by the renin–angiotensin–aldosterone system (RAAS) [98]. Unfortunately, human data are contradictory and the differences between species, e.g., in the enzymes that metabolize arachidonic acid, call into question the relevance of the rodent studies for practice [43]. In cardiovascular [61] and inflammatory diseases [62, 63], a beneficial role of n-3 PUFAs has been described. Similarly, some studies have documented a positive effect of n-3-PUFAs in kidney disease. Therapeutic application of 3 g/day n-3 PUFAs in addition to RAAS inhibition significantly increased the percentage of > 50% reduction in baseline proteinuria in patients with IgA-nephritis compared to RAAS blockade alone [64]. A recent meta-analysis confirmed that n-3 PUFA-enriched diet for more than 24 weeks could help to reduce proteinuria in patients with type 2 diabetes nephropathy [99]. In pediatric patients with type 1 diabetes, n-3 PUFA supplementation in the form of capsules improved clinical disease progression parameters including urinary albumin to creatinine ratio [100]. In pediatric kidney transplant recipients, plasma EPA was negatively associated with metabolic risk factors and blood pressure [101].

Most clinical studies have focused on fortifying the daily diet with a certain amount of n-3 PUFAs, but controlling the exact ratio of n-6 to n-3 fatty acids and the individual amounts of important fatty acids has been difficult [102]. Thus, validation parameters are needed and have been developed, like the analysis of fatty acids in red blood cells (RBCs), which reflects medium-term fatty acid intake [103]. In addition, the molecular mechanisms of how dietary interventions alter disease progression have been challenging to elucidate, especially due to limitations in tissue availability for molecular studies. Due to these methodological difficulties, there are hardly any evidence- or even consensus-based strategies for clinical application of nutritional interventions [104]. During the next couple of years however, technological advances will overcome this issue. Thus, ultra-high-performance liquid chromatography-tandem mass spectrometry has already been used to establish reference ranges for pro-inflammatory and pro-resolving lipid mediators [105]. In addition, the possibility to perform single-cell RNA sequencing from kidney biopsies [106] and urinary cells [107] will give important new insights. Future nutritional intervention studies should therefore include not only “therapeutic endpoints” but also mechanistic analyses.

## Drugs targeting lipid metabolism in kidney disease

In recent years, there has been a growing number of pharmacologically active substances developed to restore lipid metabolism balance in the kidneys. Various tabular overviews on drugs counteracting lipotoxicity in kidney disease have been published [108]. Here, only a few exemplary substances which impact upon mechanisms described in this article will be discussed. Thus, substances targeting oxysterol binding protein like 7 (OSBPL7), which increases ATP binding cassette subfamily A member 1 (ABCA1) transporter expression, have shown promising initial results in animal models of proteinuric kidney disease by reducing podocyte damage through enhanced cholesterol efflux [109]. Cyclodextrin is another substance that promotes cholesterol efflux by forming inclusion complexes with cholesterol at the membrane surface. Cyclodextrin has been shown to reduce glomerular cholesterol content and lipid accumulation in experimental models of Alport syndrome and adriamycin-induced nephropathy [110]. Fenofibrate, which activates PPAR-α [111], and astragaloside IV, which downregulates CD36/FAT [27], also reduce lipid accumulation. In addition, modulation of sphingolipid metabolism using the sphingosine-1-phosphate receptor antagonist fingolimod (FTY720) reduced albuminuria and the excretion of TNF-α in a rodent model of diabetic kidney disease [112]. The SGLT2 inhibitor JNJ39933673, which targets the sterol regulatory element-binding protein-1c (SREBP-1c) signaling pathway, reduced lipid accumulation, blood pressure, and albuminuria in leptin-receptor deficient mice [113]. Similar effects have been described for ipragliflozin [114]. The inhibition of ferroptosis by various drugs with the ability to act as radical scavengers, including promethazine and rifampicin, is another new therapeutic approach that has already yielded promising results in mouse AKI [63]. An overview of the pharmacologically active substances discussed in this article, including the most important study results, can be found in Table 2.

**Table 2:** Pharmacologically active substances / drugs relevant for kidney lipid metabolism and oxidative stress in mice and rats

Research organism	Drug	Relevant study results	Source
*Mice*	Unnamed compounds A and G	Increase the expression of ABCA1 and thereby cholesterol-efflux, leading to reduced podocyte injury/proteinuria in models of Alport syndrome/Adriamycin-induced nephropathy	[109]
	Cyclodextrin	Increases cholesterol efflux by the formation of cholesterol/cyclodextrin inclusion complexes at the membrane surface; reduces glomerular cholesterol content and lipid accumulation in models of Alport syndrome/Adriamycin-induced nephropathy	[110]
	Fenofibrate	Activates PPAR-α, thereby increasing fatty acid oxidation/reducing lipid accumulation in models of glomerular damage after high fat diet as well as tubulointerstitial injury	[111]
	Astragaloside IV	Downregulates CD36 / FAT in mesangial cells; reduces lipid accumulation and oxidative stress	[27]
	JNJ39933673	SGLT2 inhibitor; targets SREBP-1c signaling pathway; reduces GFR, albuminuria, blood pressure, and lipid accumulation	[113]
	Ipragliflozin (and others)	SGLT2 inhibitor; reduces GFR, albuminuria, blood pressure, serum triglycerides, and lipid accumulation; reduces ectopic lipid deposition and ER stress in a model of obesity-related kidney damage	[114]
	Rifampicin and promethazine	Radical scavengers, protective in mouse AKI	[63]
*Rats*	Fingolimod (FTY720)	Modulates the sphingolipid metabolism; reduces albuminuria and the excretion of TNF-α in a rat model of diabetic kidney disease	[112]

*ABCA1*, ATP binding cassette subfamily A member 1; *PPAR*-*α*, peroxisome proliferator-activated receptor alpha; *CD36*/*FAT*, cluster of differentiation 36/fatty acid translocase; *SGLT2*, sodium glucose transporter 2; *SREBP*-*1c*, sterol regulatory element-binding protein-1; *GFR*, glomerular filtration rate; *ER*, endoplasmic reticulum; *TNF*-*α*, tumor necrosis factor-alpha

## Conclusion and outlook

Impaired lipid metabolism is closely linked to the pathophysiology of many kidney diseases, including typical childhood diseases such as MCD/FSGS and neonatal AKI, but also those that may have their origin in a poor metabolic state in childhood, such as diabetic kidney disease, or finally those that can occur at any age, such as membranous nephropathy. Lipid accumulation, lipid peroxidation, and oxidative stress, as well as altered synthesis and signaling of lipid mediators, have been identified as important damage mechanisms. Given the high prevalence of CKD worldwide, it is of great socioeconomic importance to explore the mechanisms that predispose an individual to kidney disease later in life. In 2018, the American Academy of Pediatrics published a policy statement “Advocacy for Improving Nutrition in the First 1000 Days to Support Childhood Development and Adult Health” [115]. In general, the interplay of dietary fats during early childhood and dysregulation of kidney lipid metabolites and their contribution toward predisposition to kidney disease is increasingly recognized. Oxidative stress, which is closely linked to impaired kidney lipid metabolism, occurs at a young age in special risk groups such as obese children and premature infants. Genetic variability in the key regulators of kidney lipid metabolism could also contribute to individual susceptibility to certain kidney diseases and should be investigated further.

In terms of prevention, further studies are needed to establish evidence-based recommendations for the optimal composition of lipids in infant diet and the use of antioxidants during infancy. In particular, the potential of anti-inflammatory and antioxidant treatment to prevent “programmed” kidney disease in special risk groups like premature infants should be further investigated. Steadily increasing technical options to carry out multi-omics analyses from the smallest quantities of biological material will allow new insights from patient samples. Deep phenotyping of lipid metabolism in kidney physiology and pathophysiology will help to identify new therapeutic targets and eventually pave the way for new medications in manifest kidney disease.

## Supplementary Information

Below is the link to the electronic supplementary material.

Graphical abstract (PPTX 502 KB)
