# Supplementary material for: Kidney lipid metabolism: impact on pediatric kidney diseases and modulation by early-life nutrition
Source: Pediatr Nephrol. 2024 Nov 27;40(6):1839–52. doi: 10.1007/s00467-024-06595-z (PMC12031794; doi:10.1007/s00467-024-06595-z)
Supplement: Supplementary file 1 — Graphical abstract (PPTX 502 KB) [file 467_2024_6595_MOESM1_ESM.pptx]

## Slide 1
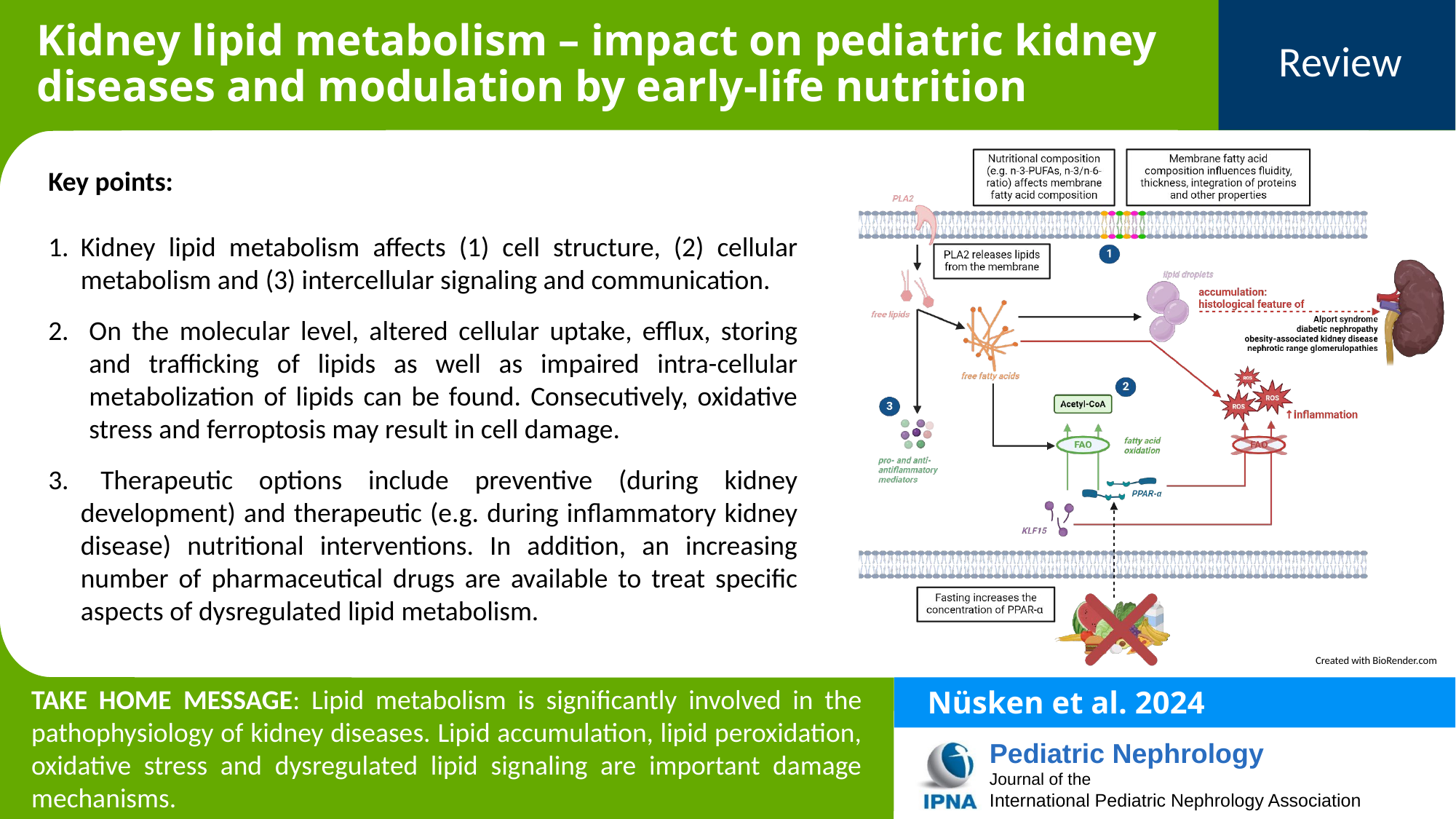

Kidney lipid metabolism – impact on pediatric kidney diseases and modulation by early-life nutrition
Key points:
1.	Kidney lipid metabolism affects (1) cell structure, (2) cellular metabolism and (3) intercellular signaling and communication.
On the molecular level, altered cellular uptake, efflux, storing and trafficking of lipids as well as impaired intra-cellular metabolization of lipids can be found. Consecutively, oxidative stress and ferroptosis may result in cell damage.
3. 	Therapeutic options include preventive (during kidney development) and therapeutic (e.g. during inflammatory kidney disease) nutritional interventions. In addition, an increasing number of pharmaceutical drugs are available to treat specific aspects of dysregulated lipid metabolism.
Created with BioRender.com
TAKE HOME MESSAGE: Lipid metabolism is significantly involved in the pathophysiology of kidney diseases. Lipid accumulation, lipid peroxidation, oxidative stress and dysregulated lipid signaling are important damage mechanisms.
Nüsken et al. 2024
